# The spectrum of neurodevelopmental, neuromuscular and neurodegenerative disorders due to defective autophagy

**DOI:** 10.1080/15548627.2021.1943177

**Published:** 2021-08-19

**Authors:** Celine Deneubourg, Mauricio Ramm, Luke J. Smith, Olga Baron, Kritarth Singh, Susan C. Byrne, Michael R. Duchen, Mathias Gautel, Eeva-Liisa Eskelinen, Manolis Fanto, Heinz Jungbluth

**Affiliations:** a Department of Basic and Clinical Neuroscience, IoPPN, King’s College London, London, UK; b Institute of Biomedicine, University of Turku, Turku, Finland; c Randall Division of Cell and Molecular Biophysics, Muscle Signalling Section, King’s College London, London, UK; d Wolfson Centre for Age-Related Diseases, King’s College London, London, UK; e Department of Cell and Developmental Biology, University College London, London, UK; f Department of Paediatric Neurology, Neuromuscular Service, Evelina’s Children Hospital, Guy’s & St. Thomas’ Hospital NHS Foundation Trust, London, UK; g Molecular and Integrative Biosciences Research Programme, University of Helsinki, Helsinki, Finland

**Keywords:** autophagy, congenital disorders of autophagy, cellular trafficking; neurodegenerative disorders, neurodevelopmental disorders

## Abstract

Primary dysfunction of autophagy due to Mendelian defects affecting core components of the autophagy machinery or closely related proteins have recently emerged as an important cause of genetic disease. This novel group of human disorders may present throughout life and comprises severe early-onset neurodevelopmental and more common adult-onset neurodegenerative disorders. Early-onset (or congenital) disorders of autophagy often share a recognizable “clinical signature,” including variable combinations of neurological, neuromuscular and multisystem manifestations. Structural CNS abnormalities, cerebellar involvement, spasticity and peripheral nerve pathology are prominent neurological features, indicating a specific vulnerability of certain neuronal populations to autophagic disturbance. A typically biphasic disease course of late-onset neurodegeneration occurring on the background of a neurodevelopmental disorder further supports a role of autophagy in both neuronal development and maintenance. Additionally, an associated myopathy has been characterized in several conditions. The differential diagnosis comprises a wide range of other multisystem disorders, including mitochondrial, glycogen and lysosomal storage disorders, as well as ciliopathies, glycosylation and vesicular trafficking defects. The clinical overlap between the congenital disorders of autophagy and these conditions reflects the multiple roles of the proteins and/or emerging molecular connections between the pathways implicated and suggests an exciting area for future research. Therapy development for congenital disorders of autophagy is still in its infancy but may result in the identification of molecules that target autophagy more specifically than currently available compounds. The close connection with adult-onset neurodegenerative disorders highlights the relevance of research into rare early-onset neurodevelopmental conditions for much more common, age-related human diseases.

**Abbreviations:** AC: anterior commissure; AD: Alzheimer disease; ALR: autophagic lysosomal reformation; ALS: amyotrophic lateral sclerosis; AMBRA1: autophagy and beclin 1 regulator 1; AMPK: AMP-activated protein kinase; ASD: autism spectrum disorder; ATG: autophagy related; BIN1: bridging integrator 1; BPAN: beta-propeller protein associated neurodegeneration; CC: corpus callosum; CHMP2B: charged multivesicular body protein 2B; CHS: Chediak-Higashi syndrome; CMA: chaperone-mediated autophagy; CMT: Charcot-Marie-Tooth disease; CNM: centronuclear myopathy; CNS: central nervous system; DNM2: dynamin 2; DPR: dipeptide repeat protein; DVL3: disheveled segment polarity protein 3; EPG5: ectopic P-granules autophagy protein 5 homolog; ER: endoplasmic reticulum; ESCRT: homotypic fusion and protein sorting complex; FIG4: FIG4 phosphoinositide 5-phosphatase; FTD: frontotemporal dementia; GBA: glucocerebrosidase; GD: Gaucher disease; GRN: progranulin; GSD: glycogen storage disorder; HC: hippocampal commissure; HD: Huntington disease; HOPS: homotypic fusion and protein sorting complex; HSPP: hereditary spastic paraparesis; LAMP2A: lysosomal associated membrane protein 2A; MEAX: X-linked myopathy with excessive autophagy; mHTT: mutant huntingtin; MSS: Marinesco-Sjoegren syndrome; MTM1: myotubularin 1; MTOR: mechanistic target of rapamycin kinase; NBIA: neurodegeneration with brain iron accumulation; NCL: neuronal ceroid lipofuscinosis; NPC1: Niemann-Pick disease type 1; PD: Parkinson disease; PtdIns3P: phosphatidylinositol-3-phosphate; RAB3GAP1: RAB3 GTPase activating protein catalytic subunit 1; RAB3GAP2: RAB3 GTPase activating non-catalytic protein subunit 2; RB1: RB1-inducible coiled-coil protein 1; RHEB: ras homolog, mTORC1 binding; SCAR20: SNX14-related ataxia; SENDA: static encephalopathy of childhood with neurodegeneration in adulthood; SNX14: sorting nexin 14; SPG11: SPG11 vesicle trafficking associated, spatacsin; SQSTM1: sequestosome 1; TBC1D20: TBC1 domain family member 20; TECPR2: tectonin beta-propeller repeat containing 2; TSC1: TSC complex subunit 1; TSC2: TSC complex subunit 2; UBQLN2: ubiquilin 2; VCP: valosin-containing protein; VMA21: vacuolar ATPase assembly factor VMA21; WDFY3/ALFY: WD repeat and FYVE domain containing protein 3; WDR45: WD repeat domain 45; WDR47: WD repeat domain 47; WMS: Warburg Micro syndrome; XLMTM: X-linked myotubular myopathy; ZFYVE26: zinc finger FYVE-type containing 26

## Introduction

Genetic defects affecting cellular pathways with fundamental biological functions are often associated with extensive and not infrequently lethal human multisystem disorders. These conditions are often named after the organelle/mechanisms involved and/or the most striking pathological abnormality seen on microscopy, and include ciliopathies, congenital disorders of glycosylation, cellular trafficking, mitochondrial, as well as glycogen and lysosomal storage disorders.

Congenital disorders of autophagy have been recently introduced as a group of novel human multisystem disorders [1] due to defects in primary elements of the autophagy pathway and closely associated proteins. The number of conditions included within this novel diagnostic category is rapidly increasing, suggesting disorders affecting an important mechanism of human disease that may be individually rare but not uncommon as a group. The degree of multisystem involvement may also point at multiple or ubiquitously essential roles of the proteins implicated. Besides its considerable clinical relevance, the concept of congenital disorders of autophagy may also crucially inform basic autophagy research: For example, the clinical signatures of individual disorders may highlight the common involvement of organs where the role of autophagy has not been fully explored yet, or, alternatively, suggest links with other cellular signaling pathways (for example, regulated cell death pathways [2]) and/or non-canonical roles of the autophagy proteins implicated that may be worth exploring. Moreover, although some of the individual conditions included within the group of congenital disorders of autophagy may be exceedingly rare, it is increasingly recognized that many of the proteins involved play an important role in much more common neurodegenerative disorders, including amyotrophic lateral sclerosis (ALS), dementia and Parkinson disease (PD) [3–7].

In the following review, we will outline the key features of the currently recognized congenital disorders of autophagy, and emphasize their overlap with other multisystem disorders, in particular cellular trafficking, mitochondrial and lysosomal storage disorders. We will highlight where such overlap may reflect close links between the respective pathways involved, and/or non-canonical roles of the primary autophagy proteins implicated. The emerging links between early-onset neurodevelopmental and adult-onset neurodegenerative disorders will be highlighted as a particularly exciting area for future research. Considering the often prominent (neuro)developmental phenotypes and the predilection for certain (neuronal) tissues in the congenital disorders of autophagy, we will outline what is currently known about the role of autophagy in embryonic, in particular neuronal, development and organ maintenance. Lastly, we will summarize currently available animal models of congenital disorders of autophagy, and prospects and limitations of therapy development.

## The autophagy pathway and its intersection with other cellular pathways.

Autophagy is a process of “self-eating” utilized by the cell to degrade material not suitable for degradation in the proteasome, for example, larger organelles such as mitochondria.

**Figure 1.**
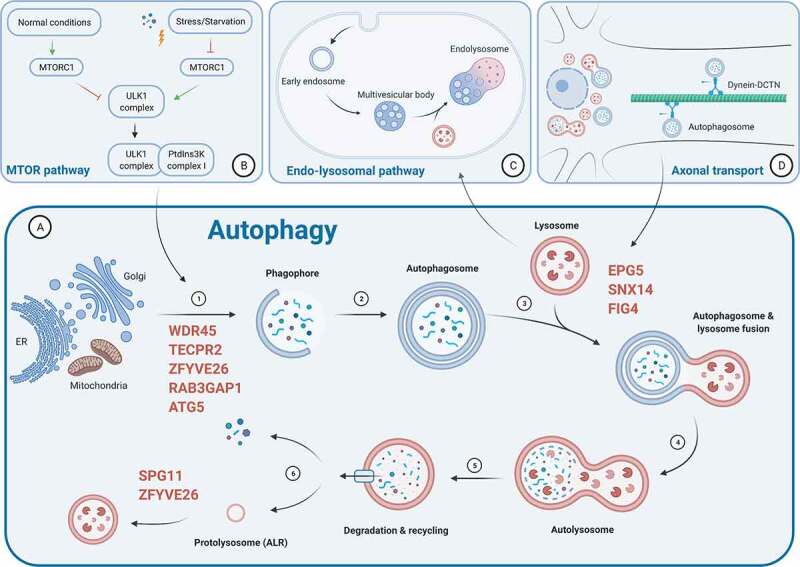
The autophagy pathway and its relation to other intracellular regulatory and trafficking pathways. (**A**) Schematic representation of the autophagy pathway and the key steps involved, ranging from phagophore formation utilizing lipid membranes from various donor compartments (such as ER, Golgi and mitochondria), autophagosome formation, autolysosomal fusion and cargo degradation, and, finally, autophagic lysosomal reformation (ALR). Gene mutations can disturb any (and often multiple) part(s) of the complex autophagic machinery; the proteins most commonly implicated in the congenital disorders of autophagy are indicated in red, in relation to the part of the autophagy pathway affected. Close relations to the MTOR pathway (**B**), the endo-lysosomal pathway (**C**) and (neuronal) axonal transport (**D**) emphasize that any genetic defect primarily affecting these intricately linked cellular processes may cause clinical presentations very similar to those concerning the primary autophagy machinery. Along similar lines, primary disturbances of other cellular processes and structures essential for the normal functioning of autophagy (for example, disturbances of lipid metabolism affecting the membrane sources required for phagophore formation, or of the glycosylation of autophagy proteins) may have similar biological and clinical consequences. Figure created with BioRender.com.

Autophagy can be divided by type – macroautophagy, microautophagy and chaperone-mediated autophagy (CMA) – and by target. These 3 different forms of autophagy all result in degradation of substrates in the lysosome, but differ in the mode of delivery: In microautophagy, the lysosomal membrane engulfs substrates directly [8]. In CMA, chaperones recognize soluble proteins bearing a specific pentapeptide motif for delivery to lysosomes, and then import them into the lysosomes via LAMP2A (lysosomal associated membrane protein 2A) [9]. Macroautophagy (hereafter autophagy) is the currently best-characterized form of autophagy, and, through the coordination of a host of specialized proteins, involves the formation of a specialized organelle, the autophagosome, and the degradation of its cargo by lysosomes. The molecular mechanisms that lead to the formation of the autophagosome and its subsequent fusion with the lysosome are well characterized (and illustrated in Figure 1). The proteins involved are encoded by 31 currently known autophagy-related (*ATG*) genes in mammals that were mostly discovered through genetic screens in yeast [10–12]. The autophagy pathway is highly conserved throughout evolution. The unique organelle of autophagy, the autophagosome, is formed from a phagophore. There are conflicting data regarding the origin of this structure, but the consensus is that it is derived, at least partially, from the endoplasmic reticulum (ER) [13–16].

MTOR (mechanistic target of rapamycin kinase) is the negative master regulator of autophagy [17]. The Ser/Thr kinase MTOR forms a complex with RPTOR (regulatory associated protein of MTOR complex 1), MLST8 (MTOR associated protein, LST8 homolog), AKT1S1 (AKT1 substrate 1) and DEPTOR (DEP domain containing MTOR interacting protein), termed MTORC1 [18–22]. The MTORC1 complex is activated by interaction with the GTP-binding protein RHEB (Ras homolog, mTORC1 binding) in response to growth factors or high levels of amino acids [23–25]. Vice versa, AMP-activated protein kinase (AMPK) inactivates MTORC1 by phosphorylation of RPTOR or TSC2 (TSC complex subunit 2) [26]. Hence, MTORC1 functions as a sensor of cellular nutrient and energy levels. The inhibitory effect of MTORC1 on autophagy was first demonstrated by the phosphorylation and consequent inactivation of the ULK1 complex comprising the protein kinase ULK1 (unc-51 like autophagy activating kinase 1) and interacting proteins ATG13 and RB1CC1 (RB1 inducible coiled-coil 1) [27–29]. More recently, it was revealed that the inhibitory effect of MTORC1 is manifold and acts on various stages of the autophagy process, as it also inactivates class III phosphatidylinositol 3-kinase (PtdIns3K), WIPI2 (WD repeat domain, phosphoinositide interacting 2), UVRAG (UV radiation resistance associated) and RUBCNL (rubicon like autophagy enhancer), which function in the formation of the nucleation membrane, phagophore elongation and fusion of the autophagosome and lysosome [30–33]. Optimal functioning of the MTORC1 pathway is thus of utmost importance for the autophagy pathway and, not unexpectedly, both processes are therefore closely interlinked [34,35]. The MTOR pathway has been implicated in a myriad of diseases such as metabolic disorders, cancer and neurodegenerative disease, the details of which go beyond the scope of this review (for a comprehensive review on MTOR signaling in disease, see [36]).

Under starvation conditions, MTORC1 is inhibited and the active ULK1 complex triggers autophagy by activating the PtdIns3K complex comprising the phosphatidylinositol 3-kinase subunit PIK3C3 (phosphatidylinositol 3-kinase catalytic subunit type 3), adaptor protein PIK3R4 (phosphoinositide-3-kinase regulatory subunit 4), regulator BECN1/Beclin-1, assembly factor NRBF2 (nuclear receptor binding factor 2) and accessory protein ATG14. ATG14 recruits the complex to the site of phagophore formation [37]. The resulting pool of phosphatidylinositol-3-phosphate (PtdIns3P) recruits proteins that drive formation of the phagophore, which recruits lipids from a donor membrane compartment to eventually extend into a double-membrane vesicle [38], highlighting the close links between autophagy and lipid, in particular phosphoinositide metabolism. Recent research has shown that ATG9 plays a crucial role in the lipid transfer needed for this phagophore extension. ATG9 is a lipid scramblase that translocates phospholipids from the cytoplasmic leaflet to the lumenal leaflet of the phagophore [39]. Multiple sites of phagophore formation and thus sources of membrane lipids have been suggested such as the ER, Golgi apparatus, endosomal vesicles and mitochondria [40,41]. It is also suggested that particular membrane contact sites between the ER and other organelles such as mitochondria are a necessary environment for phagophores to be able to appear and extend [14,41].

Recent work has shown that phosphatidylinositol-5-phosphate (PtdIns5P) can also drive phagophore formation and may be able to recruit proteins characterized as PtdIns3P-binding proteins. PtdIns3P is required for autophagy in conditions of amino acid starvation, whereas PtdIns5P is required in glucose deprivation [42], indicating that, despite shared and consistent core elements, there may be subtle differences in the molecular autophagy machinery employed under different stimuli. WIPI proteins [43] and ZFYVE1 (zinc finger FYVE-type containing 1) [38] are PtdIns3P-binding proteins recruited to the phagophore. WIPI2 recruits another complex formed of ATG12–ATG5-ATG16L1, which acts like an E3 ligase, mediating conjugation of ubiquitin-like proteins of the ATG8 family to phosphatidylethanolamine in the phagophore membrane [44]. The best-studied mammalian ATG8-family protein is LC3B, a key autophagy adaptor whose lipidation is facilitated by other proteins acting as E1- and E2-like enzymes [45]. The E2-like enzyme ATG3 specifically targets curved membranes, potentially favoring LC3B lipidation at the highly curved rim of growing phagophores [46]. LC3B is with a few exceptions [47,48] a reliable marker of autophagic structures [49].

Beyond the proteins that drive formation of the membrane structures unique to autophagy, several specialized cargo receptors deliver substrates to the phagophore. These cargo receptors include SQSTM1 (sequestosome 1), NBR1 (NBR1 autophagy cargo receptor), CALCOCO2 (calcium binding and coiled-coil domain 2) and OPTN (optineurin) and are critical for effective clearance of autophagy substrates. Mutations in both *SQSTM1* [50,51] and *OPTN* [52] are associated with ALS, with evidence of accumulation of both proteins in tissue samples from many types of neurodegenerative disorders [53,54]. The cargo receptors bind specifically to ubiquitinated substrates and to LC3 in the forming autophagosome. Although autophagy is often considered a largely nonselective process, it is now clear that there are many examples of selective autophagy mediated by autophagy cargo receptors [55].

Double-membraned autophagosomes, the result of phagophore expansion around its cargo and subsequent closure, can bear markers of early and late endosomes as well as lysosomes, suggesting a maturation process facilitated by multiple fusion events [56–58] and intricate links between autophagy and endosomal and lysosomal pathways. The final stage of autophagy is usually described as the fusion of the autophagosome and lysosome to create a hybrid organelle, the autolysosome. However, some studies suggest that autophagosomes may also deliver cargo into lysosomes via temporarily and spatially limited (“kiss and run”) interactions [59], possibly depending on circumstances and/or cell types. Whatever the precise mechanisms leading to this point, autophagic substrates are then degraded by lysosomal enzymes. As a result, lysosomal storage disorders will almost invariably also result in defective autophagy [60]. As with the initiation and maturation of autophagosomes, the final steps of autophagy involve specialized machinery. Fusion of autophagosomes and lysosomes is dependent on a SNARE complex formed by the autophagosomal SNARE STX17 (syntaxin 17), Qbc SNARE SNAP29 (synaptosome associated protein 29) and the lysosomal R-SNARE VAMP8 (vesicle associated membrane protein 8) [61]. A host of other proteins are implicated in tethering autophagosomes to lysosomes, including the homotypic fusion and protein sorting (HOPS) complex [62], TECPR1 (tectonin beta-propeller repeat containing 1) [63] and EPG5 (ectopic P-granules autophagy protein 5 homolog) [64].

Autophagosome-lysosome fusion represents the most obvious convergence of the specialized autophagy pathway and the multi-tasking endo-lysosomal system, and any disruption to lysosomal function (or indeed any other element of the complex machinery involved) may impair this crucial stage of autophagy. While both pathways differ in their starting location and cargo, with endosomes usually forming at the plasma membrane to engulf extracellular substrates and autophagosomes developing from the ER and carrying intracellular cargo, their convergence in the final step of lysosomal degradation of their respective cargos indicates their close interaction and cellular proximity. A key point of convergence between endosomal trafficking and autophagy is the endosomal sorting complexes required for transport (ESCRT). ESCRT proteins, among other roles, function sequentially in the formation of intralumenal vesicles at late endosomes to form multi-vesicular bodies. Recently ESCRT was demonstrated to function in autophagosome formation, specifically in phagophore closure [65], consistent with earlier observations that knockdown of several ESCRT proteins results in autophagosome accumulation [66].

Another group of proteins that facilitate the interactions between autophagy and the endocytic system are members of the RAB GTPase family, through their important roles in membrane trafficking, in particular the coordination of transient interactions with the outer membrane of target vesicles and the recruitment of “effector” proteins [67]. RAB7 in particular promotes processes critical for autophagosome-lysosome fusion [68], and is required to provide a pool of lysosomes for fusion with autophagosomes by interacting with the dynein-dynactin adaptor RILP (Rab interacting lysosomal protein) and thus preventing the migration of late endosomes/lysosomes to the cell periphery [69]. Especially in neuronal cells, a crucial interaction between autophagy and vesicular trafficking can be observed, as autophagosomes are usually formed in distal regions of the cells and, to allow fusion with late endosomes/lysosomes, need to be transported retrogradely along the axons to perinuclear regions [69–72]. Indirectly, impairment of the dynein-dynactin complex or microtubule obstructions can thus lead to secondary obstruction of the autophagy pathway.

Finally, after degradation of the autolysosomal content by lysosomal hydrolases, all molecules are recycled and transported to their destined cellular locations for repurposing. In addition, the autophagic lysosomal reformation (ALR) process is essential for the regeneration of a pool of functional lysosomes [73–76], and characterized by initial clathrin-mediated budding of the autolysosomal membrane, which then elongates and, through an intermediate protolysosomal stage, eventually matures into a functional lysosome. Of note, MTOR also regulates the ALR process, confirming the important interplay between MTOR and the autophagy pathway [74,75] not only at the initiation stage but at various points of the process.

The implication of these multiple and complex interactions for biologists and clinicians working on autophagy-related diseases is that autophagy must be considered not in isolation, but as part of the complex endomembrane system that is interlinked with a more complex intracellular trafficking machinery. A primary autophagy-associated disorder can thus be due to defects in any part of the autophagy pathway, including the ATG core machinery, adaptor proteins and other proteins that may not only play a role in autophagy but also in other important cellular processes.

## Congenital disorders of autophagy.

While autophagy has been implicated in a wide range of human diseases in a nonspecific way for many years, the recognition of primary Mendelian disorders with defective autophagy is only relatively recent. On clinical grounds, these disorders can be divided in often severe neurodevelopmental and neurological disorders with onset early in life (often referred to as “congenital disorders of autophagy” [1]) (Table 1), and neurodegenerative diseases presenting late in adulthood, often after a long symptom-free or subclinical interval (Table 2). However, as discussed in more detail below, the above distinction is somewhat artificial, as there is emerging evidence for a lifetime continuum of autophagy-associated disorders, both on the level of the individual patient and specific conditions.

**Table 1.** Early-onset neurodevelopmental and neurological disorders with defects in autophagy (“Congenital disorders of autophagy”) – selection

Condition	OMIM	Gene	OMIM	Protein	**Role in autophagy/abnormality**
**Multisystem disorders**					
Vici syndrome	242840	*EPG5*	615068	ectopic p-granules autophagy protein 5 homolog	Mediates autophagosome-lysosome fusion through its role as a RAB7 effector [64]
Younis Varon syndrome	216340	*FIG4*	609390	FIG4 phosphoinositide 5-phosphatase	Regulates synthesis and turnover of PtdIns(3,5)P_2_ [267]
Warburg Micro syndrome	600118	*RAB3GAP1*	602536	RAB3 GTPase activating protein catalytic subunit 1	Role in autophagosome formation [201]
**Neurodegeneration with Brain Iron Accumulation (NBIA)**					
Beta propeller-associated neurodegeneration (BPAN)	300894	*WDR45/WIPI4*	300526	WD repeat domain 45	Phagophore/autophagosome formation [92]
**Cerebellar ataxias**					
SCAR20	616354	*SNX14*	616105	sorting nexin 14	Mediates autophagosome-lysosome fusion [107]
SCAR25	617584	*ATG5*	604261	autophagy related 5	Role in autophagic vesicle formation through conjugation to ATG12 [256]
**Spastic paraplegias**					
SPG11	604360	*SPG11*	610844	SPG11 vesicle trafficking associated, spatacsin	Autolysosome recycling via ALR [110]
SPG15	270700	*ZFYVE26*	612012	zinc finger FYVE-type containing 26	Role in autophagosome formation and autophagosome-lysosome fusion; autolysosome recycling via ALR [111]
SPG49	615031	*TECPR2*	615000	tectonin beta-propeller repeat containing 2	Putative role in early autophagosome generation by scaffolding at ER exit sites [106]

**Table 2.** Adult-onset neurodegenerative disorders with defects in autophagy – selection

Condition	OMIM	Gene	OMIM	Protein	**Role in autophagy/abnormality**
**Amyotrophic lateral sclerosis (ALS)**					
FTDALS1	105550	*C9orf72*	614260	C9orf72	Role in autophagosome maturation [261]
ALS2	205100	*ALS2*	606352	alsin Rho guanine nucleotide exchange factor ALS2	Localizes to autophagosomes [244] and loss leads to autophagosome accumulation [245]
FTDALS3	616437	*SQSTM1*	601530	sequestosome 1	Autophagy receptor required for aggrephagy [296]
ALS4	602433	*SETX*	608465	senataxin	Role in autophagosome maturation [282]
ALS5	602099	*SPG11*	610844	SPG11 vesicle trafficking associated, spatacsin	Autolysosome recycling via ALR [110]
ALS11	612577	*FIG4*	609390	FIG4 phosphoinositide 5-phosphatase	Regulates synthesis and turnover of PtdIns(3,5)P_2_ [267]
ALS12	613435	*OPTN*	602432	optineurin	Selective autophagy receptor implicated in aggrephagy, mitophagy, and xenophagy [278]
FTDALS6	613954	*VCP*	601023	valosin containing protein	Role in autophagy initiation [290] and in mitophagy [291]
FTDALS7	614696	*CHMP2B*	609512	charged multivesicular body protein 2B	Part of the ESCRT-III complex [263], with a role in mitophagy-specific phagophore closure [65]
**Parkinson disease (PD)**					
PARK2	600116	*PRKN/PARK2*	602544	parkin RBR E3 ubiquitin protein ligase	Induces mitophagy in concert with PINK1, and within a E3 ubiquitin ligase complex [155]
PARK6	605909	*PINK1*	608309	PTEN induced kinase 1	Induces mitophagy by recruitment of PRKN [155]
PARK20	615530	*SYNJ1*	604297	synaptojanin 1	Autolysosome recycling via ALR [110]

Congenital disorders of autophagy often have a recognizable “clinical signature,” characterized by 1) prominent neurological and neuromuscular phenotypes, 2) a combination of developmental and degenerative abnormalities evolving over time and 3) variable degrees of multiorgan involvement. They may affect any component associated with the autophagy machinery, and the degree of multisystem involvement in particular may also point at the multiple roles of the proteins implicated, not all necessarily primarily autophagy-related. On clinical grounds, congenital disorders of autophagy (Table 1) can be subdivided into those with prominent multisystem involvement, brain iron accumulation, hereditary spastic paraplegias and cerebellar ataxias, although there is considerable clinical overlap between those entities.

**Figure 2.**
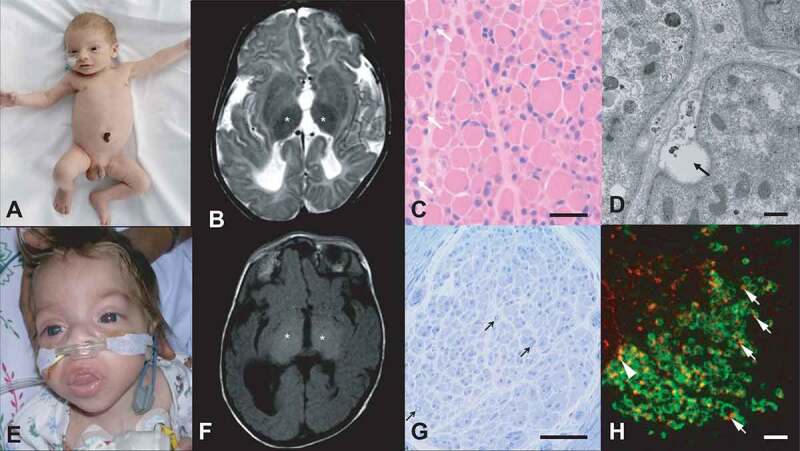
Key clinical and pathological features of EPG5-related Vici syndrome, the paradigmatic congenital disorder of autophagy. Patients of Turkish (**A**) and Indian (**E**) descent with hypopigmentation relative to ethnic background. Although neurological findings may be subtle at an early age (**A**), more severely affected patients may show coarse facial features suggestive of a storage disorder (**E**) and neurological deterioration from early infancy. Cataracts are common. Thalamic changes characterized by low signal on T2- (**B**) (asterisks) and high signal on T1-weighted brain images (**F**) (asterisks) may be observed in a proportion of patients and have also been reported in some lysosomal storage disorders. On light microscopy, (**C**) muscle biopsy findings are characterized by increased variability in fiber size and the presence of numerous internalized and central nuclei (arrows), resembling centronuclear myopathy and X-linked myotubular myopathy (scale bar: 50 μm). On the ultrastructural level (**D**), in skeletal muscle there are numerous vacuoles and evidence of ongoing exocytosis (arrow) (scale bar: 500 nm). A peripheral neuropathy characterized by marked reduction of myelinated fibers (arrows) on sural nerve biopsy stained with Toluidine Blue (**G**) has been reported in few patients (scale bar: 50 μm). On confocal immunohistochemistry of EPG5-mutated fibroblasts treated with bafilomycin A_1_ (**H**), compared to normal fibroblasts where numerous LC3-positive autophagosomes are found engulfed by the LAMP1-positive vesicular structures (data not shown), relatively small LC3-positive puncta (in red) only sporadically colocalize with LAMP1 (in green, arrowhead shows colocalization), with many isolated LC3-positive puncta (arrows). In addition, in EPG5-mutated fibroblasts the LC3 signal is seen mainly at the rim of LAMP1-positive structures rather than centrally. These findings are indicative of an autophagosome-lysosome fusion defect (scale bar: 5 μm).

The paradigmatic disorder of defective autophagy is Vici syndrome (Figure 2), one of the most extensive human multisystem disorders reported to date and characterized by callosal agenesis, cataracts, cleft palate, cardiomyopathy, combined immunodeficiency, hypopigmentation, acquired microcephaly and failure to thrive [77–79]. Vici syndrome was initially reported in 1988 by Dionisi-Vici and colleagues in two brothers and subsequently attributed to recessive mutations in *EPG5* [78,79]. EPG5 is ubiquitously expressed and has a critical role in autophagosome-lysosome fusion and, probably, other intracellular vesicular fusion events [64,80]. There may be variability of protein levels and, consequently, the clinical phenotype in particular in association with the many splice mutations identified [81]. Subtle clinical manifestations, in particular an apparently higher incidence of cataracts, vitiligo and certain cancers [82–85], have been observed in heterozygous *EPG5* mutation carriers, suggesting either pathogenic haploinsufficiency or a toxic gain-of-function effect over time in cases where a truncated EPG5 protein is not subjected to intracellular decay. Although other features are less consistently associated, virtually any organ system – including thyroid, lungs, liver and kidneys – may be affected in Vici syndrome patients, in keeping with the ubiquitous expression of the EPG5 protein and its fundamental biological importance. Interestingly, on the level of specific organs, *EPG5*-related Vici syndrome may feature both congenital defects as well as disease acquired later in life (for example, congenital heart defects *and* cardiomyopathy later in life, structural central nervous system [CNS] thyroid agenesis *and* hypothyroidism in a normally formed thyroid), emphasizing the crucial role of normal autophagy for both organ development and maintenance. The degree of cardiac and immune involvement are the main determinants of prognosis.

While essentially a multisystem disorder, neurological aspects are most prominent in *EPG5*-related Vici syndrome and comprise both neurodevelopmental and neurodegenerative aspects [86]. In addition to the structural CNS abnormalities and the acquired microcephaly already mentioned, severe developmental delay, early-onset epilepsy, sensorineural deafness and movement disorders are frequently observed [79]. Evidence for peripheral neuropathies with similarities to the neuropathy reported due to mutations in the EPG5-interactor RAB7 [87] has been reported in isolated patients. A skeletal myopathy is consistently associated with *EPG5*-related Vici syndrome and is evidenced by variable degrees of hypotonia and weakness, and mild creatine kinase elevations [88].

Following the genetic resolution of Vici syndrome in 2013, a number of additional early-onset neurodevelopmental and neurological disorders (Table 1 and S1) are now attributed to mutations affecting primary components of the autophagy machinery downstream of MTORC1, including other multisystem disorders such as Warburg Micro syndrome (WMS) and Younis Varon syndrome due to recessive mutations in *RAB3GAP1* (RAB3 GTPase activating protein catalytic subunit 1) [89] and *FIG4* (FIG4 phosphoinositide 5-phosphatase) [90], respectively, beta-propeller protein-associated neurodegeneration (BPAN) due to X-linked dominant *(de novo)* mutations in *WDR45* (WD repeat domain 45) [91,92], cerebellar ataxia associated with autosomal-recessive mutations in *SNX14* (sorting nexin 14) and in *ATG5* [93], and three forms of hereditary spastic paraparesis (HSPP), SPG11 (spastic paraplegia 11), SPG15 and SPG49 caused by autosomal-recessive mutations in *SPG11* (SPG11 vesicle trafficking associated, spatacsin), *ZFYVE26* (zinc finger FYVE-type containing 26) and *TECPR2* (tectonin beta-propeller repeat containing 2), respectively [1,94,95].

Clinically, among the multisystem disorders, Warburg Micro syndrome (WMS) ([96,97] for review of clinical and genetic heterogeneity) shares a number of features with Vici syndrome, in particular severe mental retardation, corpus callosum hypoplasia, (acquired) microcephaly, congenital cataracts, optic atrophy and seizures, a phenotypical overlap likely to be explained by the recently described close molecular links between EPG5 and RAB3GAP1 [98], one of the causative proteins implicated in this genetically heterogeneous condition. Interestingly, like other autophagy-related disorders, WMS typically shows a biphasic course, characterized by spastic paraparesis evolving over time following an initial presentation with profound hypotonia. Younis Varon syndrome (for review [90]) is a severe neurodevelopmental disorder with early lethality and vacuolar changes in various tissues, including neurons and muscles, due to autosomal-recessive mutations in *FIG4*. Variably associated features, in particular corpus callosum abnormalities, congenital heart defects, cardiomyopathy and hearing impairment, show some overlap with Vici syndrome. Autophagy defects have been demonstrated in *FIG4*-mutated Schwann cells [99]. As in other autophagy-related disorders, depending on specific genotype, the associated clinical spectrum is wide, ranging from severe early-onset neurodevelopmental disorders to forms of Charcot-Marie-Tooth (CMT) disease (CMT4J) [100] and ALS (ALS11) [101]. Corresponding to the human phenotype, Fig4 null mice have a multisystem disorder with neurodegenerative features [100].

*WDR45*-associated BPAN (previously also known as “Static Encephalopathy of Childhood with NeuroDegeneration in Adulthood”, or SENDA) [102] belongs to the wider group of conditions characterized by Neurodegeneration with Brain Iron Accumulation (NBIA), predominantly affecting the basal ganglia. The condition initially presents with global developmental delay, seizures and variable additional neurological features in childhood before a relentlessly progressive course characterized by dystonia, parkinsonism and cognitive decline develop from adolescence onwards [103].

Cerebellar ataxias are also common among primary autophagy disorders: In addition to global developmental delay, seizures and progressive cerebellar atrophy, children with *SNX14*-related ataxia (SCAR20) [104] often demonstrate additional multisystem features suggestive of a storage disorder. *ATG5-*related SCAR25 is another recessively inherited ataxia recently attributed to mutations in a core autophagy component in a single family [105].

Another common manifestation are hereditary spastic paraplegias: *SPG11-* and *ZFYVE26-*related HSPP share signs of early-onset spasticity, frequently with a consistent combination of features (referred to as “Kjellin syndrome”) comprising intellectual impairment, pigmentary retinopathy, cerebellar dysfunction and, variably, parkinsonism. Autosomal-recessive spastic paraplegia 49 due to homozygosity for a *TECPR2* founder mutation in Jewish Bukharian families is a complex multisystem disorder characterized by distinct dysmorphic features, severe central apneas, progressive intellectual impairment, spastic paraplegia and ataxia.

Although the genes and proteins implicated in these congenital disorders of autophagy are likely to have multiple functions, a connection to the autophagy pathway has been clearly established, with SPG49-associated TECPR2 and BPAN-associated WDR45 linked to the phagophore/autophagosome formation stages [91,92,106], SCAR20-associated SNX14 and EPG5 related to autophagosome-lysosome fusion [107,108] and the spastic paraplegia proteins SPG11 and ZFYVE26 [109] implicated at multiple levels, including the recycling of autolysosomes [110,111]. WDR45/WIPI4 (the mammalian homolog of *C. elegans* EPG-6), the protein mutated in BPAN, belongs to the family of WD40 proteins which, through their highly stable and symmetrical beta-propeller superstructure, play a crucial role in facilitating the assembly of multiprotein complexes; with regards to autophagy, they specifically interact with ATG2 and ATG9 to facilitate autophagosome formation and elongation. EPG5, the protein mutated in Vici syndrome, is an example of a RAB7 effector directly involved in autophagy. EPG5 is recruited to lysosomes by GTP-bound RAB7 and facilitates autophagosome-lysosome fusion by interacting with the SNARE complex and LC3 [64]. EPG5 knockdown results in the formation of enlarged perinuclear vesicles that are positive for markers of early and late endosomes, as well as autophagy markers [64]. SNX14, the protein mutated in SCAR20, is a member of the sorting nexin family with an ability to bind membrane phosphatidylinositol residues, a quality of likely relevance to autophagosome/lysosome formation where distinct organelle phospholipid signatures ensure specificity of fusion events [107]. ZFYVE26 and SPG11, the gene products of *ZFYVE26* and *SPG11* implicated in two forms of hereditary spastic paraplegia, respectively, form a complex targeted to the lysosome and are also probably functionally closely related. A role in membrane trafficking for ZFYVE26 is suggested by its ability to bind PtdIns3P [112]. With regards to the autophagy pathway and at least partly mediated through its interactions with the BECN1-UVRAG-RUBCN (rubicon autophagy regulator) complex, ZFYVE26 has been specifically implicated in autophagosome formation, autophagosome-lysosome fusion and, in concert with SPG11, the recycling of autolysosomes via the ALR pathway [111]. The role of defective TECPR2 implicated in SPG49 is not fully resolved but may involve impairment of early autophagosome generation due to reduced scaffolding at ER membrane exit sites [106].

It is likely that as yet unresolved neurodevelopmental disorders with overlapping clinical features (in particular those combining multisystem involvement, cerebellar signs and spastic paraparesis) will be attributed to mutations affecting additional components of the complex autophagy machinery in future, bearing in mind, however, that some of these mutations may also be antenatally lethal.

### Neurodevelopmental features

Congenital disorders of autophagy often show a combination of (neuro)developmental and (neuro)degenerative features evolving over time, supporting a role of autophagy in both embryonic (neuronal) development and maintenance later in life.

Autophagy genes such as *EPG5* have been implicated in human and murine stem cell development [113], and observations both in congenital disorders of autophagy but also in relation to other autophagy-associated genes suggest additional roles further downstream in neuronal differentiation. Another autophagy gene in which defects have been shown to impede neurodevelopment is WDFY3 (WD repeat and FYVE domain containing 3), a large PtdIns3P-binding and scaffold protein functioning in aggrephagy, the removal of aggregated proteins by autophagy [114], and in mitophagy, the selective degradation of mitochondria by autophagy [115]. Loss of WDFY3 has been reported as a risk factor in patients with autism spectrum disorder (ASD) [116] where early brain overgrowth is a common feature [117]. Early brain overgrowth is also recapitulated in *Wdfy3*-mutant mice, which feature increased brain size due to altered neural progenitor proliferation [118]. Interestingly, a missense mutation in *WDFY3* has been found to have the opposing effect and cause hereditary primary microcephaly [119] and reduced brain size in humans and a corresponding *Drosophila* model, suggesting that *WDFY3* plays a general role in determining correct brain size, probably through attenuation of WNT signaling through removal of DVL3 (disheveled segment polarity protein 3) aggregates [119]. Indeed, Le Duc et al. showed that mutations in *WDFY3*, which putatively cause haploinsufficiency, lead to macrocephaly and nonspecific mild neurodevelopmental delay, while mutations in the PH-domain of *WDFY3* lead to microcephaly in affected patients [120]. Another reported function of WDFY3 supporting a role in neuronal differentiation is its implication in the formation of axonal tracts in the brain and spinal cord of mice [121].

Accumulating evidence suggests that autophagy has a role in various stages of neurodevelopment: In the early stages, such a role has been demonstrated by knockout of *Ambra1* (autophagy and beclin 1 regulator 1) in mice, causing neural tube defects in these animals [122]. At the later stage of neurite formation, increased autophagy by cell-specific knockout of *Mtor* causes suppressed proliferation in GABAergic progenitor cells, leading to reduced cortical interneurons in mice [123]. In addition, knockdown of *Atg7* by siRNA causes abnormally elongated axons in primary rat cortical neurons, while autophagy induction by rapamycin has a suppressing effect on axon growth [124]. Mice with a loss of function mutation in *Wdr47* (WD repeat domain 47) display increased autophagic flux. WDR47 is a negative regulator of autophagy that modulates microtubule dynamics and controls neuronal polarity [125]. In these mice, a decrease in proliferation of progenitor cells during embryonic development causes decreased neurogenesis, which results in absence of all major axonal tracts, including the anterior commissure (AC), the hippocampal commissure (HC), and the corpus callosum (CC), implying a general role of WDR47 in axonal outgrowth *in vivo*. Along similar lines, decreased autophagic activity due to mutations in *Atg16l1* increases the size of the CC in mice [126]. An opposing effect for autophagy disruption is observed by brain-specific knockout of *Atg9a* in mice, where defective neurite overgrowth as well as dysgenesis of the CC and the AC are prominent features. Unexpectedly, the authors reported that primary murine neurons with a depletion of *Atg7* and *Atg16l1* do not display the same defects [127], suggesting that the effects observed in *atg9a* knockout animals may not be primarily due to autophagy interruption, but disrupted non-canonical functions of *Atg9a*. A similar observation has been made in *epg5* knockout animals, in which non-canonical dysfunction may be an important contributor to the observed decrease in CC size [128], as demonstrated by the deceleration in endocytosis and the delay in endocytic recycling in these animals [129]. Ablation of *Ulk1* and *Ulk2*, required for parallel fiber formation of granule cells, represents a similar scenario: Ablation of both proteins blocks neurite formation in primary murine granule cells *in vitro* [130] and, if specifically ablated in the CNS, causes defective axonal pathfinding and defasciculation in the CC, AC, corticothalamic axons and thalamocortical axons [131]. However, none of these defects are recapitulated in *Atg7* and *Rb1cc1* (RB1-inducible coiled-coil 1) mutants, suggesting a mechanism related to a disturbance of non-canonical roles rather than to the primary autophagy defect.

Interestingly, there is also some indication that the effect of autophagy on neurite and synapse formation and remodeling may be dependent on the specific neuronal subtype. Stavoe et al. [132] reported that mutations in six autophagy genes in *C. elegans* (*atg-9, atg-13, epg-8, igg-1, atg-2* and *unc-104*) lead to longer nociceptors while HSN, RIA, DA9, RIB, and NSM neurons do not show a comparable phenotypic alteration. The same study reported that disruption of 18 different autophagy genes in *C. elegans* is not only affecting neurite formation, but also synapse formation, the subsequent step in neurodevelopment. Mutant animals exhibit impaired vesicle clustering and reduced active zone formation [132], comparable to earlier findings in *Drosophila melanogaster* where depletion of *Atg1/unc-51* (*ULK1* in mammals) leads to defects in active zone formation associated with impaired neurotransmitter release [133]. The development of neuromuscular junctions, the muscle innervating synapses, in *Drosophila melanogaster* is also impaired in *Atg1, Atg2, Atg6*, and *Atg18* mutants, while overexpression of *Atg1* increases the number of synaptic boutons [134]. A similar phenotype is observed in mouse models, in which motor neuron-specific ablation of *Atg7* leads to 37% larger neurons, denervation of motor endplates and reduced neurotransmission [135].

Lastly, autophagy is also implicated in spine pruning, the final step of neuronal development that eliminates excess dendrites. Mice with heterozygous mutations in genes encoding MTOR inhibitors *Tsc1* (TSC complex subunit 1) and *Tsc2* (TSC complex subunit 2) in layer V pyramidal neurons exhibit higher numbers of spines in the cortex due to defective spine pruning as a consequence of low autophagic activity [136]. It can thus be speculated that, in the absence of critical autophagy factors, improper pruning of axons and synapses by microglia, i.e. in a non-cell-autonomous manner, causes the observed abnormalities in axon formation and neurite outgrowth mentioned above.

In conclusion, autophagy has been implicated in virtually all steps of neurodevelopment. However, its effects are not consistent and can either have a promoting or inhibiting effect on neuronal development and differentiation, depending on the developmental process and the cell type. Importantly, some studies suggest that although the ablation of autophagy genes gives rise to neurodevelopmental defects, the observed phenotypes may be caused by non-canonical functions of these genes rather than a direct disruption of autophagic pathways.

### Neurological and neurodegenerative features

As outlined in more detail above, congenital disorders of autophagy often show a characteristic evolution of neurological features over time, with neurological symptoms such as spasticity, ataxia, dystonia and epilepsy often evolving on the background of a preexisting neurodevelopmental disorder. In addition, an increasing number of adult-onset neurodegenerative disorders have recently been attributed to primary defects in the autophagy pathway (Table 2 and S1), supporting a role of autophagy not only in normal neuronal development and differentiation but also in maintenance of the nervous system.

In general terms, the large size and the high energy demands of neuronal cells represent a special challenge for homeostasis. Neurons as highly specialized cells are maintained mostly throughout the whole lifespan of an organism, and therefore rely heavily on housekeeping mechanisms that safeguard the integrity of organelles and proteostatic processes. More specifically, evidence for the crucial role of autophagy in physiological neuronal maintenance was originally derived from conditional knockout of the key autophagy genes *Atg5* and *Atg7* in the mouse brain [137,138], resulting in axonal swellings and ultimately degeneration of cerebellar Purkinje cells and hippocampal pyramidal neurons. Along similar lines, neuron-specific knockout of *Rb1cc1* leads to accumulation of ubiquitin-positive aggregates and degeneration of Purkinje cells in mice [139], whereas heterozygous deletion of *Becn1* shows loss of synapses and dendrites of the hippocampus [139,140]. Since then, much attention has been invested in further elucidating the role of autophagy in neurons and glial cells, in relation to their specific function and their very distinct polarized morphology: Synapse development and maintenance, generation and turnover of autophagosomes, their subsequent transport along the microtubules inside the long dendritic and axonal projections and concurring consumption rely on precise spatial and temporal regulation and coordination [141], resulting in complex compartmentalization of neuronal autophagic mechanisms [142]. Other peculiar features of the nervous system adding further levels of complexity are the transcellular exchange of debris from neurons to glial cells [143], and, as a metabolically highly demanding tissue, its heavy reliance on mitochondrial integrity and function. Research concerning aging-related neurodegenerative conditions has therefore focused on the selective autophagic digestion of dysfunctional mitochondria (or mitophagy) as well as removal of aggregation-prone proteins (or aggrephagy) [141].

A peculiar feature of the congenital disorders of autophagy is their predilection for certain parts of the nervous system, in particular the cerebellum, long white matter tracts and peripheral neurons. Why exactly certain neuronal subtypes such as cerebellar Purkinje cells are predominantly affected in these conditions has yet to be clarified, but it is conceivable that their complex architecture and high metabolic activity could be the reason for their selective vulnerability. In addition, autophagy plays a crucial role in the homeostasis of axons, as supported by the axonal pathology observed in autophagy-deficient mouse models, and by the prominent involvement of brain white matter tracts and peripheral nerves, structures consisting mainly of axonal projections, in humans with congenital disorders of autophagy. Autophagosomes in particular constitutively form in the distal end of axons and are transported retrogradely to the cell soma for degradation [144,145], and any defect disturbing this essential mechanism is likely to result in neuronal pathology. The latter hypothesis is supported by the observation that mutations affecting proteins primarily involved in axonal (including autophagosomal) transport cause similar phenotypes as primary autophagy disorders, probably due to secondary effects on correct autophagosomal positioning [146,147].

In addition to these basic considerations and in a more clinical context, autophagy has been associated with adult-onset neurodegenerative disorders in several ways (for review [148],), nonspecifically through its complex interactions with the potentially toxic protein aggregates implicated in these conditions, and, specifically, through primary genetic mutations directly or indirectly affecting components of the autophagy machinery (summarized in Table S1): Autophagy plays a role, for example, in the removal of SNCA (synuclein alpha) in PD [149,150], misfolded proteins in ALS [52,151], mutant HTT (huntingtin; mHTT) in Huntington disease (HD) [114,152] and intracellular MAPT/TAU tangles in Alzheimer disease (AD) [153,154], but at the same time these targets of autophagic digestions may impair normal autophagic flux and functioning through their inherent toxicity. While rare, primary genetic causes in particular of PD also indicate a role of defective autophagy, for example autosomal-recessive mutations in *PINK1* and *PARK2*, encoding two proteins that act in concert to designate damaged mitochondria for mitophagic digestion and thus play an important role in mitochondrial quality control [155]. Mutations in *LRRK2* have been associated with dominant forms of PD and have been demonstrated to affect the proper functioning of CMA [156], but LRRK2’s precise role in autophagy currently still awaits resolution [157]. Of note, heterozygosity for mutations in the lysosomal enzyme GBA (glucosylceramidase beta), the gene recessively mutated in Gaucher disease (GD), is the most common genetic risk factor for PD and associated with autophagic impairment, emphasizing the intimate crosstalk between autophagic and lysosomal pathways [158,159].

The role of autophagy in ALS is illustrated through a small proportion of familial ALS cases due to pathogenic variants in the autophagy receptors SQSTM1 [160], OPTN [161], and UBQLN2 (ubiquilin 2) [162], all of which facilitate cargo recruitment into phagophores through their interactions with LC3. Homozygous mutations in *SQSTM1* lead to a congenital absence of the autophagy receptor and cause a childhood-onset neurodegenerative condition with a phenotype comprising ataxia/cerebellar syndrome, parkinsonism, and cognitive decline [163]. Different forms of ALS (and/or of frontotemporal dementia [FTD] with overlapping clinical features) have also been linked to heterozygous dominant mutations in *VCP* encoding valosin-containing protein (FTDALS6) [164] (also implicated in inclusion myopathy with Paget disease of the bone and FTD [165]), *CHMP2B* encoding charged multivesicular body protein 2B (FTDALS7) [166], and *GRN* encoding progranulin [167]. Although the roles of these proteins are clearly multiple, VCP and CHMP2B both function in autophagy, while progranulin is important for both autophagosome and lysosome function [168]. Interestingly and corresponding to what has been observed with *SQSTM1*, while heterozygous mutations in *GRN* lead to frontotemporal degeneration later in life, homozygous *GRN* mutations cause a neurodevelopmental disorder with features similar to a lysosomal storage disorder or a neuronal ceroid lipofuscinosis (NCL11) [169].

Although not a primary component of the autophagy machinery and likely to be associated with multiple roles, *C9orf72*, the gene most commonly associated with ALS, has also been linked to autophagy [170,171]. ALS-associated hexanucleotide repeat expansions in the *C9orf72* gene are translated into dipeptide repeat (DPR) proteins, which are prone to aggregate and ultimately lead to neurodegeneration. Interestingly, *EPG5* has been shown to be a modifier of this DPR toxicity, emphasizing again the role of autophagy in neurodegenerative diseases [172].

It is clear from *SQSTM1* and *GBA* that the age of onset and phenotype severity are related to the genetic burden, with homozygous mutations causing rare childhood-onset neurodegenerative syndromes while heterozygous mutations are associated with more common late-onset neurodegenerative disorders such as ALS and PD. Simply put, the mutation dosage may very well determine the phenotype due to the time it takes for aggregates to accumulate. This also suggests that the key to effective therapeutic intervention in classical late-onset neurodegenerative disorders may lie in earlier treatment. In addition, family studies in rare early-onset autophagy disorders may play a valuable role in the identification of additional genetic risk factors for neurodegenerative disorders, or vice versa.

### Neuromuscular features

*EPG5*-related Vici syndrome, the paradigmatic disorder of defective autophagy, shows a consistently associated myopathy, on the histopathological level characterized by increased fiber size variability, increased (central) nucleation, fiber type disproportion with predominance of type 1 fibers, vacuolization, increased glycogen storage and variable mitochondrial abnormalities, including respiratory chain enzyme abnormalities [79,88,173]. The *EPG5*-associated myopathy shows considerable histopathological overlap with primary vacuolar myopathies, centronuclear myopathy (CNM), X-linked myotubular myopathy (XLMTM), and glycogen storage disorders (GSDs), and it is not unexpected that also in these disorders both primary and secondary defects of autophagy have now been critically implicated (Table 3): Danon Disease and X-linked myopathy with excessive autophagy (MEAX), the most common vacuolar myopathies, are due to X-linked mutations in *LAMP2* [174] and *VMA21* (vacuolar ATPase assembly factor VMA21) [175], two genes encoding a lysosomal membrane component and a subunit of the lysosomal vacuolar-type H^+^-ATPase important for lysosomal acidification, respectively. While in both conditions, the primary defect thus likely originates in the lysosome, histopathological features of marked autophagic buildup and variable degrees of exocytosis are identical to what has been observed in *EPG5*-related Vici syndrome. “Retrograde” abnormalities of autophagosome formation and autophagosome-lysosome fusion have also been observed in Pompe Disease [176], a GSD secondary to deficiency of lysosomal GAA (alpha glucosidase), emphasizing the close connection between lysosomal and autophagic abnormalities. The marked similarities between the *EPG5*-related myopathy and CNM/XLMTM are particularly intriguing, considering that many of the major genes implicated in these conditions – *MTM1* (myotubularin 1), *DNM2* (dynamin 2) and *BIN1* (bridging integrator 1) – play an important role in intracellular membrane trafficking and have been linked with defective autophagy in human cells and animal models [147,177,178]. In addition, MTM1 plays a specific role in the regulation of PtdIns3P levels [179], an important substrate at the phagophore initiation stages (see above).

**Table 3.** Neuromuscular disorders with defects in autophagy – selection

Condition	OMIM	Gene	OMIM	Protein	**Role in autophagy/abnormality**
**Vacuolar myopathies**					
Danon disease	300257	*LAMP2*	309060	lysosomal associated membrane protein 2	“Retrograde” autophagic abnormalities secondary to impaired autolysosomal fusion [174]
X-linked myopathy with excessive autophagy (XMEA)	310440	*VMA21*	300913	vacuolar ATPase assembly factor VMA21	“Retrograde” autophagic abnormalities secondary to abnormal lysosomal acidification [175]
**Glycogen storage disorders (GSD)**					
Glycogen storage disease (GSD) type 2	232300	*GAA*	606800	alpha glucosidase	“Retrograde” abnormalities of autophagosome formation and autophagosome-lysosome fusion secondary to abnormal lysosomal glycogen storage [176]
**Centronuclear myopathies**					
X-linked myotubular myopathy (XLMTM)	310400	*MTM1*	300415	myotubularin 1	Regulator of PtdIns3P pool; disconnection between fasting and autophagy initiation [178]; failure of ATG machinery to dissociate from PAS site [295]
Centronuclear myopathy (CNM)	160150	*DNM2*	602378	dynamin 2	Autophagic buildup and delayed autophagosomal maturation in DNM2 mouse models [147]

In contrast to *EPG5*-related Vici syndrome, a skeletal myopathy has not been reported in any of the other congenital disorders of autophagy yet. This may reflect a genuine absence or, more likely considering the prominent expression of genes such as *WDR45* in skeletal muscle, an overlooked aspect due to the overwhelming severity of neurological and other multisystem features. Of note, all genes/proteins implicated in the congenital disorders of autophagy are also expressed in skeletal muscle, although for *TECPR2* so far this has only been demonstrated at the gene/RNA level and not yet at the protein level.

### Differential diagnosis and overlap with other early-onset neurological multisystem disorders

Congenital disorders of autophagy show a wide range of clinical and histopathological features, and other multisystem disorders with neurological involvement, as well as acquired or inherited primary neurological or neuromuscular disorders, ought to be considered in the differential diagnosis (Table 4). The overlap with other neurological multisystem disorders may reflect close links between the autophagy pathway and the organelles implicated in the respective multisystem disorder, or, alternatively, a genuine dual role of the protein implicated in the respective congenital disorder of autophagy. Examples for clinical similarities based on the close links of the autophagy pathway with other pathways are lysosomal storage and mitochondrial disorders, which often have similar clinical features, reflective of the lysosome as the endpoint of autophagy, and of autophagy (in its specialized form of mitophagy) as an important mitochondrial quality control mechanism.

**Table 4.** Differential diagnosis of congenital disorders of autophagy

Group of conditions	Molecular basis for clinical overlap with autophagy disorders
Lysosomal storage disorders	Close connections between autophagic and lysosomal pathways; Retrograde autophagic abnormalities in lysosomal storage disorders; Anterograde lysosomal abnormalities in autophagy disorders
Mitochondrial disorders	Secondary mitochondrial dysfunction due to defective mitophagy
Glycogen storage disorders	Retrograde autophagic abnormalities due to abnormal glycogen storage
Vesicular trafficking disorders	Close connection between autophagic and endosomal/endocytic trafficking pathways; Involvement of mutated genes in multiple pathways
Glycosylation disorders	Glycosylation important for normal functioning of certain autophagy proteins; Role of glycoconjugates in autophagy induction; Role of autophagy in turnover of free cytosolic glycans
Ciliopathies	Role of ciliary pathways in controlling autophagy; Association between ciliary dysfunction and impaired autophagy

Although the primary defects in congenital disorders of autophagy do not reside directly in the lysosome, they may mimic some of the clinical features of lysosomal storage disorders (for review [180,181]), for example the coarse facial appearance seen in *SNX14*-related cerebellar ataxia and, occasionally, *EPG5*-related Vici syndrome. The latter group of patients may also develop thalamic changes on brain MRI similar to those seen in disorders of primary lysosomal origin [79]. In addition to the intracellular accumulation of undigested macromolecules in lysosomes, a hallmark feature of lysosomal storage disorders, secondary deficits in upstream autophagy pathway have been frequently documented [60,182]. Impaired autophagic flux and accumulation of autophagosomes in *EPG5*-related Vici syndrome closely resembles the histopathological findings in lysosomal storage disorders such as GM1 gangliosidosis, Niemann-Pick disease type C1 (NPC1), and the neuronal ceroid lipofuscinoses (NCLs) [182,183]. Along similar lines, patients with bi-allelic *SNX14* mutations share both clinical and pathological features of lysosomal storage (such as enlarged lysosomes) and autophagic (such as reduced autophagic flux) disorders, suggesting that deficits in both autophagy and lysosomal function may contribute to certain features of the clinical phenotype in *SNX14*-related ataxia such as prominent cerebellar involvement and Purkinje cell loss [107].

In addition to lysosomal storage disorders, many clinical aspects of congenital disorders of autophagy also resemble mitochondrial disorders. Progressive impairment of the mitochondrial form and function is the hallmark feature of monogenic mitochondrial disorders, a heterogeneous group of nearly 50 diseases caused by mutations in 228 protein-encoding nuclear DNA genes and 13 mitochondrial DNA (mtDNA) genes [184,185]. The mitochondrial morphology and/or respiratory chain defects caused by these mutations primarily affect energy production, leading to neuropathological and neuromuscular manifestations of mitochondrial disorders, which preferentially affect the striated muscles and nervous system [186,187]. Abnormal mitochondrial ultrastructure as well as altered respiratory chain enzyme function with predominant skeletal myopathy diagnosed in patients suggest secondary mitochondrial dysfunction as an important mechanism in *EPG5*-related Vici syndrome, and may lead to the differential diagnosis of one of the mitochondrial disorders [187,188]. Since mitochondrial homeostasis critically depends on downstream pathways that mediate selective removal of damaged mitochondria by autophagy and lysosomal degradation, secondary mitochondrial dysfunction and defective mitophagy are common pathological findings in autophagy and lysosomal storage disorders. Evidence of impaired mitophagy has long been recognized in patient samples and disease models of a variety of different autophagy disorders [189]. Deficits in mitochondrial form and function are also critical contributors to progressive neurodegeneration, as observed in *PINK1*- and *PRKN*-related early-onset PD [190]. Defective mitophagy induced by defects in the PINK1-PRKN pathway, which normally involves a complex interplay of PINK1-dependent phosphorylation and PRKN-mediated ubiquitination events on the outer mitochondrial membrane resulting in the selective sequestration of ubiquitinated mitochondria within autophagosomes, leads to accumulation of damaged mitochondria and subsequent neuronal cell death [191,192]. Defective mitophagy has also been observed in association with mutations in *OPTN* encoding optineurin [193].

Another important group of conditions that show marked clinical overlap with disorders of autophagy are disorders of intracellular (vesicular) trafficking, probably reflective of the fact that some of the proteins implicated in congenital disorders of autophagy, such as EPG5, do have genuinely multiple roles in both autophagy but also other, in particular endosomal/endocytic trafficking pathways [194]. Indeed, it may be very difficult to distinguish which of the phenotypical expressions of a mutated protein may be due to its role in autophagic or other closely related vesicular trafficking processes. Based on the assumption that multisystem disorders linked in the same molecular pathways do share a recognizable “clinical” signature, a reverse search on Human Dysmorphology databases applying the key features of *EPG5*-related Vici syndrome reveals indeed a number of clinically similar conditions due to mutations affecting proteins that are implicated in cellular trafficking processes in a wider sense, but that may also give rise to secondary autophagy abnormalities. For example, Marinesco-Sjogren syndrome (MSS) [195] and related disorders share cataracts, a skeletal muscle myopathy and, in some cases, sensorineural deafness with *EPG5*-related Vici syndrome, although other neurological features and the degree of multisystem involvement are usually less pronounced. Corresponding to the observed clinical overlap, impaired autophagy and nuclear abnormalities similar to those observed in primary autophagy disorders have been described in muscle tissue from SIL1-deprived mice, an animal model of MSS [195–197]; although the precise basis for this observation remains uncertain, disturbance of the autophagy pathway at the phagophore stage is one plausible hypothesis, considering the prominent role of SIL1 at the ER. Another group of conditions that show considerable overlap with *EPG5*-related Vici syndrome are Chediak-Higashi syndrome (CHS) [198] and related primary immunodeficiency syndromes, with common features of hypopigmentation, immune defects, variable neurological involvement, and, in some cases, a vacuolar myopathy. Corresponding to the observed clinical overlap, mutations in *LYST*, the causative gene, have been shown to affect lysosome size and quantity but also to cause autophagic abnormalities, although evidence for the latter has been discussed controversially [199].

The hypothesis of disorders linked in connected autophagy and trafficking pathways sharing the same “clinical signature” was recently supported by the identification of an interaction between EPG5 and RAB3GAP1, the protein mutated in Warburg Micro syndrome [200], as outlined above a clinical phenocopy of *EPG5*-related Vici syndrome. Furthermore, WMS can also be caused by mutations in *RAB3GAP2* (RAB3 GTPase activating non-catalytic protein subunit 2), RAB18 and TBC1D20 (TBC1 domain family member 20). In addition to their primary trafficking functions, both RAB3GAP2 and RAB18 also play an important role in autophagosome formation, while TBC1D20 is needed for autophagosome maturation [200–203]. The clear link between the autophagy pathway and cellular trafficking due to mutations in those genes may thus explain the observed clinical similarities between WMS and *EPG5*-related Vici syndrome.

There are a number of other human multisystem disorders, which show considerable overlap with congenital disorders of autophagy, including glycogen storage disorders, congenital disorders of glycosylation, ciliopathies and peroxisomal disorders.

Increased glycogen is common on muscle biopsies from patients with *EPG5*-related Vici syndrome, and, in conjunction with occasionally observed organomegaly, may give rise to the suspicion of one of the glycogen storage disorders [204], another group of conditions that is closely linked with both abnormal autophagy and lysosomal pathology [205]. The accumulation of autophagic debris, glycogen-containing lysosomes as well as exocytosed vesicles observed in those muscle biopsies share common pathological features with the severe metabolic myopathy observed in one of the GSDs, Pompe Disease [84,206].

Clear links are also emerging between congenital disorders of glycosylation [207,208] and the autophagy pathway: For example, various key autophagy proteins such as SNAP29 and BECN1 require post-translational O-GlcNAc glycosylation for their proper functioning [208,209], suggesting that any primary defect in these pathways will also have downstream functional consequences on autophagy. Furthermore, glycoconjugates have also been described as inducers of autophagy by decreasing the activity of the MTOR pathway [105], and probably play an important role in autophagosome formation, considering their localization on the ER and leading edges of the phagophore. Lastly, the interaction between the two pathways is bi-directional, as autophagy regulates the turnover of free glycans, which can accumulate in the cytosol [207,209]. Given the number of interactions between autophagy and glycosylation as outlined above, the strong clinical overlap with shared features of developmental delay, failure to thrive, neurological and neuromuscular abnormalities, and cardiac involvement [79,207], is thus not unexpected.

Ciliopathies, the summary term for a variety of diseases caused by impairment of the formation or function of primary cilia [210–213], are another clinically similar group of disorders that may feature multiorgan involvement but also neurological and structural CNS abnormalities including agenesis of the corpus callosum. Recently strong bi-directional interactions between autophagy and cilia have been described, in which ciliary pathways control autophagy and conversely autophagy is important for regulating ciliogenesis [213,214]. Both inducers (e.g., IFT20) and suppressors (e.g., OFD1) of ciliogenesis can be degraded through autophagy, stressing its influence on the formation of cilia and its potential role in the cause of ciliopathies. *Vice versa*, it has been shown that autophagy is decreased in cells with compromised cilia, possibly via activation of the MTOR pathway, again indicating their close interaction [213,214].

In conclusion, the vast clinical overlap between the above-mentioned groups of disorders and congenital disorders of autophagy is not very surprising, giving the clear mechanistic links between the different pathways (summarized in Table 4). Many of the proteins causing these diseases have multiple roles in several cellular pathways, making it sometimes difficult to pinpoint the specific cause of a disease or a particular symptom. Understanding autophagy and its crosstalk with other cell type-specific homeostatic pathways, in particular membrane and vesicle trafficking, lysosomal pathways and autolysosome consumption [215], will probably help to unravel additional aspects of autophagy specific to neurons, and explain their specific vulnerability to defects affecting these processes.

## Animal models of congenital disorders of autophagy

Animal models emulating human pathologies allow insights into disease mechanisms on cell biological level, which are often hard or impossible to gather otherwise.

The best-characterized animal model for Vici syndrome is the *epg5*^−/-^ mouse model. *epg5*^−/-^ mice show a clear neurodegenerative phenotype as observed in humans, however this only arises in adulthood, while the majority of Vici syndrome patients present with very early-onset neurodegeneration [79,128]. Another difference is the lack of neurodevelopmental features in the *epg5*^−/-^ mice, although they do have a reduced (but not absent) corpus callosum, a myopathy and retinitis pigmentosa [128,216]. These differences between the human and the murine phenotype suggest that caution is required when investigating complex human multisystem disorders in relevant animal models, which are however still indispensable for the development and pre-clinical testing of new therapies.

Although development of specific therapies for congenital disorders of autophagy is still at a very early stage, lessons may be learned from recent more advanced developments concerning disorders, which are not strictly part of this group but where defective autophagy is a prominent feature. For example, a number of different (including pharmacological, genetic and enzyme replacement) approaches have been investigated for XLMTM due to X-linked recessive mutations in myotubularin, a regulator of the PtdIns3P pool essential for phagophore formation in the early stages of autophagy [217,218]. Some of these approaches are currently reaching the stage of clinical application and may serve as models for therapy development in congenital disorders of autophagy.

Interestingly, there are a number of phenotypes in animal models with primary defects in autophagy for which no corresponding human phenotype has been identified yet, for example those affecting the cysteine protease ATG4, which is required for c-terminal hydrolysis of ATG8 family proteins including LC3 [219]. *ATG4B*-deficient mice show slightly abnormal cerebellar morphology and mild motor impairment [220] while a point mutation in the *ATG4D* gene has been identified as the cause of a hereditary neurodegenerative disease in Lagotto Romagnolo dogs, associated with decreased autophagic flux under basal conditions [221,222]. Another congenital disease of autophagy identified in dogs is hereditary ataxia caused by mutations in *RAB24* [223]. RAB24 functions in the clearance of autolysosomes in basal autophagy [224].

Identification of disease-causing mutations in animal models like these could help to shed more light on human disease for which no causative genes have been pinpointed yet, in particular in the context of the wealth of genetic data currently generated in the context of diagnostic next-generation sequencing.

## Neurodevelopmental and neurodegenerative disorders due to defective autophagy – a lifetime continuum of neurological disease

**Figure 3.**
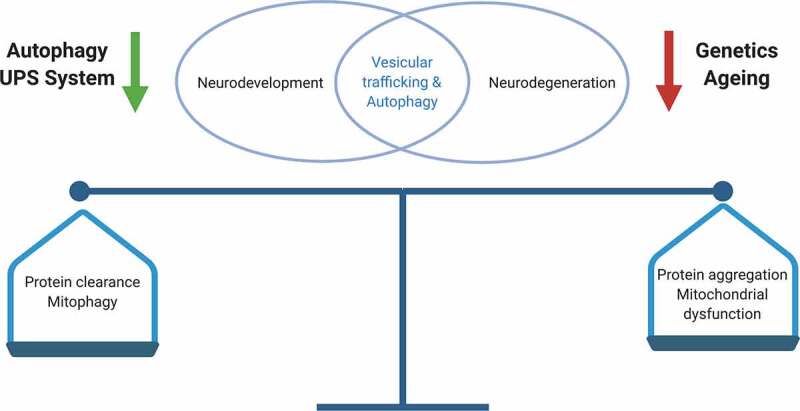
Neurodevelopmental and neurodegenerative disorders with defects in intracellular trafficking and autophagy. The accumulation of abnormal protein aggregates and defective organelles (in particular mitochondria) with age is counterbalanced by intracellular quality control mechanisms including mitophagy and aggregate removal through autophagy and/or the ubiquitin-proteasome (UPS) system. In genetic conditions impairing the effective actions of these intracellular pathways, the balance is shifted, resulting in neurodegenerative changes usually occurring later in life. Early-onset neurodevelopmental and adult-onset neurodegenerative disorders with defects in autophagy thus represent a highly interconnected spectrum of disorders associated with premature neuronal aging presenting throughout life.

As outlined in the preceding paragraphs, there is mounting and multiple clinical and molecular evidence for a continuum between congenital disorders of autophagy and adult-onset neurodegenerative disease: 1) while essentially neurodevelopmental disorders at the outset, most congenital disorders of autophagy follow a biphasic course, with a phase of accelerated deterioration following an initial period of apparent stability. Such a period of accelerated deterioration, characterized by progressive dementia, epilepsy and movement disorders (often of a Parkinsonian nature) is typical in *WDR45*-associated BPAN and *SNX14*-associated cerebellar ataxia, but also in *EPG5*-related Vici syndrome, as evidenced by rapidly progressive microcephaly and an evolving movement disorder in long-term survivors. 2) Animal models of defective autophagy show CNS features mimicking adult-onset neurodegenerative disorders, as demonstrated in a conditional *Epg5 Drosophila* knockdown showing severe retinal neurodegeneration [84], by clinical and pathological features of human ALS in the *epg5^−/-^* mouse [129], and by similar findings in a range of murine models of Atg deficiency [225]. 3) Other early-onset neurodevelopmental disorders linked in related vesicular trafficking and lysosomal pathways, for example *LYST*-related CHS [226–228] but also *SIL1*-related MSS [229], also demonstrate a higher incidence of early-onset (Parkinsonian) movement disorder. 4) Risk variants in the *EPG5* [172] but also *SIL1* gene [230] have been identified as modifiers of adult-onset neurodegenerative disorders including dementia, PD and ALS. 5) An increasing number of adult-onset neurodegenerative disorders including dementia, PD and ALS have now been linked to components of the autophagy pathway (Table S1). Taken together, these observations suggest a continuum between early-onset neurodevelopmental and adult-onset neurodegenerative disorders connected in intricately linked intracellular vesicle trafficking pathways and underline that the thorough investigation of rare genetic childhood disorders may be of relevance for the understanding of much more common age-related conditions (Figure 3).

## Therapeutic perspectives

Given the key role of autophagy in many human diseases, this fundamentally important intracellular pathway has been under intense investigation for therapeutic exploitation [148]. While in the cancer field, most attempts have focused on blocking autophagy, in neurodevelopmental and neurodegenerative disorders, the agreed consensus is that what is desirable is the stimulation rather than the blockade of autophagy. Most approaches so far have focused on neurodegenerative disorders and on targeting the regulatory signaling pathways upstream of autophagy rather than the autophagy process itself. Therapeutic approaches utilizing small molecules and focusing on upstream regulatory pathways such as the MTOR pathway can be grossly divided into those acting in an MTOR-dependent and those acting in an MTOR-independent manner. Amongst the MTOR-dependent approaches, Rapamycin (which induces autophagy through its MTOR antagonism) has been shown to ameliorate the phenotype of models for AD, PD and HD [231–233]. An alternative, MTOR-independent approach is autophagy stimulation via the AMPK pathway utilizing molecules such as trehalose [234]. However, stimulation of autophagy initiation is not always helpful and there are examples where a detrimental effect has been observed in animal models of neurodegeneration in which the clearance step is stalled [235,236]. It is also worth noting that autophagy may be protective or pathogenic depending on the disease stage [135]. Moreover, many neurodegenerative diseases but in particular neurodevelopmental disorders such as *EPG5*-related Vici syndrome, display defects in the actual autophagy cycle, leading to a slower or impeded clearance. It is reasonable to expect that this blockage is not overcome by merely increasing the levels of autophagy initiation upstream, as evidenced by the absence of any survival benefit in a large phase III trial of the putative autophagy enhancer (lithium) in patients with ALS. A plausible alternative would be to rather increase all pathways of lysosomal clearance, including autophagy and lysosomal biogenesis, controlled by the transcription factor TFEB and related members of the MiT/TFE family [237–239]. Several interventions based on *TFEB*-related gene therapy approaches have indeed been shown to be effective in numerous animal models of neurodegenerative diseases [240–243], 246–296 and in GSDs such as Pompe disease. Most therapeutic approaches applied to date modify autophagy in a very general way, and small molecules that would target the autophagy pathway more specifically and tailored to the underlying molecular defect may represent a more effective approach to neurodevelopmental and neurodegenerative disease with defective autophagy in future.

As a general point concerning late-onset neurodegenerative disorders associated with defective autophagy but without a specific causative gene, it is still uncertain if autophagy as such is normal and is just overwhelmed over time leading to issues later in life, or if, alternatively, there is a primary issue present from birth that very slowly leads to problems after decades, as supported by the observation outlined above that mutation dosage appears to determine age of onset. Whatever the underlying scenario, it is likely that in late-onset neurodegenerative disorders, the process of defective autophagy is present for many years before becoming clinically evident, suggesting a prolonged window of opportunity for therapeutic intervention. Subtle clinical signs or symptoms may be present for years (if not decades) in susceptible patients, indicating markers that may allow identification of patients for early treatment [65].

## Conclusions and outlook

Neurodevelopmental disorders with defective autophagy represent a novel and rapidly expanding group of inborn errors of metabolism. While the causative defects affect primarily the autophagy machinery and associated proteins, there is considerable overlap with other inborn multisystem disorders, in particular lysosomal disorders and those due to defects in vesicular and membrane trafficking [178]. Overlap of clinical features and communality of molecular mechanisms suggest a continuum of early-onset neurodevelopmental and adult-onset neurodegenerative disorders with defects in autophagy and intracellular trafficking throughout life. Considering that the autophagy pathway is highly amenable to pharmacological modification, the development of therapies tailored to specific underlying molecular mechanisms and the complex neuronal environment represents the ultimate goal [174].

## Supplementary Material

Supplemental Material
